# Protective Effects of Curcumin in Cardiovascular Diseases—Impact on Oxidative Stress and Mitochondria

**DOI:** 10.3390/cells11030342

**Published:** 2022-01-20

**Authors:** Fiona Frederike Cox, Angelina Misiou, Annika Vierkant, Niloofar Ale-Agha, Maria Grandoch, Judith Haendeler, Joachim Altschmied

**Affiliations:** 1 Environmentally-Induced Cardiovascular Degeneration, Clinical Chemistry and Laboratory Diagnostics, Medical Faculty, University Hospital and Heinrich-Heine-University, 40225 Düsseldorf, Germany; fiona.cox@hhu.de (F.F.C.); misiou@uni-duesseldorf.de (A.M.); annika.vierkant@hhu.de (A.V.); aleagha@hhu.de (N.A.-A.); 2 Institute for Pharmacology and Clinical Pharmacology, Medical Faculty, University Hospital and Heinrich-Heine-University, 40225 Düsseldorf, Germany; maria.grandoch@hhu.de; 3 IUF-Leibniz Research Institute for Environmental Medicine, 40225 Düsseldorf, Germany

**Keywords:** aging, atherosclerosis, cardiovascular diseases, curcumin, mitochondria, myocardial infarction, obesity, oxidative stress, risk factors

## Abstract

Cardiovascular diseases (CVDs) contribute to a large part of worldwide mortality. Similarly, two of the major risk factors for these diseases, aging and obesity, are also global problems. Aging, the gradual decline of body functions, is non-modifiable. Obesity, a modifiable risk factor for CVDs, also predisposes to type 2 diabetes mellitus (T2DM). Moreover, it affects not only the vasculature and the heart but also specific fat depots, which themselves have a major impact on the development and progression of CVDs. Common denominators of aging, obesity, and T2DM include oxidative stress, mitochondrial dysfunction, metabolic abnormalities such as altered lipid profiles and glucose metabolism, and inflammation. Several plant substances such as curcumin, the major active compound in turmeric root, have been used for a long time in traditional medicine and for the treatment of CVDs. Newer mechanistic, animal, and human studies provide evidence that curcumin has pleiotropic effects and attenuates numerous parameters which contribute to an increased risk for CVDs in aging as well as in obesity. Thus, curcumin as a nutraceutical could hold promise in the prevention of CVDs, but more standardized clinical trials are required to fully unravel its potential.

## 1. Introduction

### 1.1. Cardiovascular Diseases

Cardiovascular diseases (CVDs) are the leading cause of death worldwide. According to the World Health Organization (WHO), the almost 18 million deaths due to CVDs accounted for 32% of global deaths in 2019. This report also revealed that CVDs do not exclusively affect industrialized countries, as over three-quarters of CVD-related deaths occur in low- and middle-income countries (https://www.who.int/news-room/fact-sheets/detail/cardiovascular-diseases-(cvds) (accessed on 8 January 2022)). Older projections have already indicated that this number is expected to increase to over 23 million by 2030 [1]. In addition to being the major cause of death worldwide, CVDs also lead to a great number of chronically ill patients, and as a consequence, to an immense socio-economic burden. Thus, there is an urgent global need for efficient CVD prevention.

There are numerous established risk factors for the development and progression of CVDs. The aging process per se is a non-modifiable risk factor, as it cannot be reversed. On the contrary, other factors such as obesity, which, according to the WHO report on global health risks, is one of the major causes of ischemic heart disease [2], are modifiable, meaning that measures can be taken to change them and thereby reduce the risk for CVDs.

Many natural substances have been used in traditional medicine in many regions of the world, often for thousands of years. In this review, we will highlight the impact of curcumin on age-related cardiovascular dysfunction, adipose tissue, and obesity, as well as its protective effects in atherosclerosis and myocardial infarction.

### 1.2. Curcumin as Nutraceutical with Pleiotropic Actions

Curcumin has been used as a spice, herbal supplement, food additive, and traditional medicine in Asia for more than 4000 years. It has been widely studied with respect to many diseases and is considered to have a huge potential medical benefit. The polyphenolic compound is the major active component extracted from the rhizomes of turmeric (*Curcuma longa*). First described by Vogel and Pelletier as a “yellow coloring-matter” in 1815 [3], the pure compound was obtained in 1842 [4]. It took until 1910 before the chemical structure was identified as diferuloylmethane [5] and another 8 years before it was synthesized [6]. The effects of this nutraceutical with regard to its anti-bacterial, anti-inflammatory, anti-oxidant, and anti-cancer properties have been studied in vitro and in vivo for a long time [7,8,9,10]; additionally, numerous clinical trials addressing various human diseases have been conducted [11].

## 2. Cardiovascular Diseases and Risk Factors

### 2.1. Atherosclerosis and Myocardial Infarction

There are various types of CVDs affecting the vessels and the heart. Atherosclerosis is a complex and multi-factorial disease driven by low-grade inflammation. During disease progression, a fibrous plaque is built up on the arterial wall leading to progredient narrowing of the vessel lumen. One of the key events in the initiation of atherosclerosis, besides cholesterol deposition in the vessel walls and chronic inflammatory reactions, is endothelial dysfunction, which occurs in areas of arteries prone to plaque development [12] that are characterized by disturbed blood flow and an increase in reactive oxygen species (ROS). ROS are produced by various systems, including NADPH oxidases and the mitochondria, in endothelial as well as in smooth muscle cells [13]. Under homeostatic conditions, excessive ROS production is counteracted by anti-oxidative systems such as Glutathione (GSH), Superoxide dismutases (SODs), and Catalase, which are downregulated by various risk factors for CVDs.

Oxidative stress in the vasculature does not only affect the vascular wall but also leads to the oxidation of lipids, which in turn play a critical role in atherosclerosis development and progression [14,15,16]. Seemingly, there is interdependence between lipids and oxidative stress in atherosclerosis. This is exemplified by increased mitochondrial ROS production in low-density lipoprotein (LDL) receptor (*Ldlr*)-deficient mice [17], an established atherosclerosis model. Moreover, Apolipoprotein E (*Apoe*) knockout mice that additionally lack or have reduced levels of Manganese superoxide dismutase (SOD2), the mitochondrial isoform of SOD, and have enhanced oxidative stress display significantly increased atherosclerotic lesion formation [18].

In addition to affecting the peripheral vasculature, atherosclerosis is one of the major underlying causes of myocardial infarction (MI) and stroke [19]. MI is caused by the occlusion of a coronary artery resulting in diminished blood flow which causes ischemia. Upon reperfusion, there is a surge in ROS which causes additional damage [20]. This so-called ischemia/reperfusion (I/R) injury leads to cardiomyocyte death and at worst case is lethal. The healing process after MI involves the differentiation of cardiac fibroblasts into myofibroblasts, mechanically strong cells, which can passively participate in the contraction and form a stable scar. However, persistent activation of fibroblasts can lead to pathological fibrosis [21]. Moreover, endothelial cells in the heart are required for revascularization in the damaged area [22].

Interestingly, most, if not all, cell types in the vascular wall and the heart, which are affected in atherosclerosis and MI, depend on proper mitochondrial function. This is unquestionable for cardiomyocytes but has also been shown for endothelial cells [23] and cardiac fibroblasts [24].

### 2.2. Brown and Perivascular Adipose Tissue in Cardiovascular Diseases

The vascular wall and the heart are not the only ones to play critical roles in CVDs, a tissue that has long been regarded as being simply responsible for thermogenesis—brown adipose tissue (BAT) does as well. BAT is rich in mitochondria, which are crucial for heat production by non-shivering thermogenesis via the expression of Uncoupling protein 1 (UCP1). This transmembrane protein translocates protons through the inner mitochondrial membrane, bypassing the mitochondrial ATP synthase, which leads to increased energy expenditure and heat production. In humans, BAT is formed during early fetal development and is located in axillary, cervical, perirenal, and periadrenal regions. It is largest at birth and though its size decreases with age, BAT is still present and active in adults [25,26,27,28]. In addition to being thermogenic, BAT is involved in the regulation of energy balance and body weight, as well as in the control of glucose and lipid metabolism. Interestingly, white adipocytes, which make up white adipose tissue (WAT) as the major energy storage organ can be converted to brown adipocytes in a process called beiging [29].

A protective role for BAT in CVDs has been shown in different animal models. Multiple studies using atherosclerosis-prone mice demonstrated that activation of BAT, either by short-term cold exposure or by pharmacological interventions, accelerates the clearance of triglycerides from the plasma. These fatty acids from triglyceride-rich lipoproteins are taken up by BAT where they are subjected to mitochondrial fatty acid oxidation [30,31]. This BAT-mediated reduction of hyperlipidemia and hypercholesterolemia protects from atherosclerosis development in mice [32].

The findings obtained using the above-described animal models are supported by a study in humans that could correlate cold-induced BAT activation and lower levels of cardiovascular risk factors at baseline as well as reduced intima-media thickness and increased elasticity of the carotids after 5 years [33]. In addition, a large retrospective study, in which individuals were categorized according to the presence or absence of BAT, associated the lower prevalence of cardiometabolic disease with the presence of BAT. Moreover, the presence of BAT correlated with lower odds for several cardiovascular risk factors as well as for coronary artery and cerebrovascular disease [34].

Another distinct adipose tissue depot relevant for CVDs is the perivascular adipose tissue (PVAT). The close anatomic relationship with the vessel wall is especially suggestive of the modulating role of PVAT in vascular homeostasis. Indeed, while PVAT was for a long time assumed to be non-functional, recent studies reported endocrine and paracrine functions with the release of specific adipokines, cytokines, and chemokines. Thereby, PVAT affects inflammatory responses and also vascular functions [35]. PVAT is not homogeneous as there are clear locoregional differences. While thoracic PVAT is similar to brown adipocytes, abdominal PVAT rather resembles a WAT-like phenotype [36,37] and this morphological dissimilarity is reflected in differences in function [38].

Similar to BAT, PVAT also plays a role in the development of CVDs. However, due to the different composition of PVAT along arteries and the differences in PVAT in healthy versus obese individuals, the situation is more complicated [39]. A protective role for PVAT in CVD has been demonstrated by enhanced atherosclerosis in mice after PVAT ablation. On the contrary, the cold exposure of animals containing PVAT improved endothelial functions and inhibited atherosclerosis [40]. In addition, murine thoracic PVAT is resistant to diet-induced macrophage infiltration and might thus play a role in protecting against inflammation [36]. PVAT, similar to the endothelium, expresses endothelial Nitric oxide synthase (eNOS), which can compensate for reduced NO production due to endothelial dysfunction in aortas from hypercholesterolemic *Ldlr*-deficient mice [41]. However, diet-induced obesity in wild-type mice induced uncoupling of eNOS and increased superoxide production in PVAT [42]. Of note, a short-term high-fat diet (HFD) induces endothelial dysfunction specifically in the abdominal but not the thoracic aorta [43]. This was ascribed to differential reactions of the distinct PVAT compartments and illustrated by a decrease in unsaturated lipids only in the abdominal rather than in the thoracic PVAT [43,44]. With respect to the protective functions of PVAT, it has also been shown that Adiponectin secreted from PVAT suppresses plaque formation [45]. Along this line, continuous exogenous Adiponectin administration decreased PVAT inflammation and normalized endothelial function in rats on HFD [46].

As the differences in thoracic and abdominal PVAT reflect the different susceptibility of specific aortic sections for atherosclerosis [47], maintenance or reactivation of thoracic PVAT should also be considered as a potential option in the protection against CVDs.

### 2.3. Aging as a Non-Modifiable Risk Factor for Cardiovascular Diseases

According to the United Nations’ World Population Prospects from 2019, one in six people globally will be above the age of 65 by 2025. Data from the American Heart Association Heart Disease and Stroke Statistics show that the prevalence of CVDs in the US population increases with age in both males and females [48]. This problem is also pertinent worldwide with aging being one of the major drivers of the increase in CVDs [49]. Along these lines, aging is an independent risk factor for the development of CVD as the incidence rises steeply with advanced age [50,51].

With increasing age, several changes take place in the vasculature and heart that enhance the risk for cardiovascular events. Key vascular alterations include endothelial dysfunction which impairs vasodilatory and anti-thrombotic responses, thus favoring atherogenesis. Additionally, changes in the extracellular matrix of the vascular wall are associated with aortic stiffening and elevated systolic blood pressure. This in turn increases the left ventricular afterload finally leading to hypertrophy of the heart. In addition, remodeling of the myocardial microvasculature decreases perfusion and increases the risk for ischemia [52].

The heart and the vascular wall are not the only ones affected during the aging process, but also the different fat depots containing brown adipocytes. While a reduction in BAT size with age has been known for a long time, a comparison of pluripotent PVAT-derived adipose stromal cells (PVASC) from young and old mice revealed a decreased endothelial and brown adipogenic differentiation capacity with age. Furthermore, implantation of PVASC from old animals in the perivascular tissue of carotids after ligation injury promoted neointimal hyperplasia which was not observed when PVASC from young animals were used. In this experimental setting, human PVASC from coronary artery bypass graft patients also accelerated the thickening of the neointima. In accordance with the data obtained in mice, these cells could differentiate into various cell types, but not into brown adipocytes [53].

One hallmark of aging is cellular senescence, a state of cell cycle arrest, triggered in part by the p53/Cyclin-dependent kinase inhibitor 1A (CDKN1A or p21) pathway [54]. It has been demonstrated that the accumulation of senescent cells in the vasculature contributes to cardiovascular and metabolic diseases [55]. Cellular senescence is accompanied by a senescence-associated secretory phenotype (SASP) which can lead to a bystander effect in neighboring cells.

A major molecular change, which occurs in senescence and aging that can contribute to CVD development is oxidative stress, a shift in the cellular redox balance towards increased ROS levels. This increase in ROS is caused by an upregulation of oxidative systems, among them the enzymes mentioned before, but also the mitochondria which are the main consumers of oxygen in cells and generate superoxide as a byproduct of the electron transport chain [56]. Notably, the levels of superoxide and lipid peroxidation products were elevated in the cardiomyocytes of aged humans [57]. Interestingly, NADPH oxidase 4, another ROS generating system, is upregulated in the aging heart and is a causative factor in age-associated aortic stiffening, an independent predictor of CVDs [58,59]. The impaired mitochondrial functionality with age also explains the decrease in the functional capacity of the cardiovascular system [60] because a proper mitochondrial function is required in most resident cells of the blood vessel wall and the heart.

Oxidative stress is normally counteracted by the induction of anti-oxidative defense systems to re-establish the cellular redox homeostasis. A central player in this response is the transcription factor NFE2 like BZIP transcription factor 2 (NFE2L2), also called NRF2. NRF2 upregulates the expression of genes coding for cytosolic, nuclear, and mitochondrial enzymes essential for GSH production and regeneration as well as the ones required for ROS detoxification or the reduction of oxidized proteins [61]. Therefore, NRF2 has been termed as a master regulator of anti-oxidative responses [62]. However, NRF2 does not only activate anti-oxidative defense systems in response to oxidative stress, but also directly regulates mitochondrial respiration by modulating the availability of substrates, and thus has a profound impact on mitochondrial functions [63].

With increasing age, the levels and DNA-binding activity of NRF2 decline dramatically, not only in the liver [64,65], but more importantly in the vasculature and the heart. This age-related loss of NRF2 in the cardiovascular system is accompanied by a downregulation of numerous anti-oxidative systems and an increase in ROS [66,67]. A direct role for NRF2 in the prevention of vascular senescence in vivo has unambiguously been demonstrated in aged NRF2-deficient mice which show aggravated cellular senescence in the cerebral vasculature with concomitant induction of a SASP [68]. However, NRF2 can be reactivated in old animals leading to the normalization of hepatic GSH levels [64]. Moreover, activation of NRF2 can also counteract vascular senescence. This has been shown in vitro where activation of NRF2 protected vascular smooth muscle cells against angiotensin II-induced senescence [69] and endothelial progenitor cells (from diabetic animals) against dysfunction by suppressing senescence [70]. Even in whole animals, the impaired function of NRF2 in age-related myocardial oxidative stress can be reversed, e.g., by moderate exercise [67]. Based on these studies, the activation of NRF2 might be an interesting approach to improve cardiovascular health in the elderly.

### 2.4. Obesity as a Modifiable Risk Factor for Cardiovascular Diseases

Obesity is defined by a body-mass index (BMI) above 30, while individuals with a BMI between 25 and 29.9 are considered overweight. According to this definition, the worldwide prevalence of obesity has steadily been rising over the last 40 years and has increased more than fourfold in children and adolescents globally. This problem is not restricted to high-income countries, but is also relevant in middle- and low-income countries [71]. Furthermore, it has been suggested that simply using the BMI as a measure might lead to substantial underestimation of the problem [72].

Obesity is characterized by a massive expansion of body fat; however, it entails many other detrimental metabolic changes, e.g., an increased risk for developing T2DM. It has been estimated that obesity with increased abdominal fat accounts for 80–90% of all T2DM cases [73]. Abdominal obesity is also linked to metabolic syndrome [74], a cluster of conditions, which is characterized by dyslipidemia, impaired glucose tolerance, and high blood pressure.

The lipid alterations in metabolic syndrome include elevated serum triglycerides, total and low densitix lipoprotein (LDL) cholesterol, whereas the levels of high density lipoprotein (HDL) cholesterol are reduced. In contrast, LDL cholesterol is usually normal in T2DM patients, but the retention time in the blood is prolonged. Moreover, the LDL particles are rich in triglycerides [75]. Not surprisingly, similar alterations are found in obesity [76].

Obesity not only affects lipid levels and composition but also BAT and PVAT. While the volume of active BAT seems to be reduced in obese human subjects [77], BAT from obese mice is hypertrophic but characterized by increased inflammation and oxidative damage [78]. Although no direct measurements of PVAT volume have been made, it has been shown in several animal models that obesity entails PVAT dysfunction [42,79]. A similar observation was made in obese patients [80]. This dysfunction seems to be due to local adipose tissue inflammation, as a reduction in inflammation can restore PVAT functions in obese mice [81] but also in humans despite persistent obesity [82].

Another commonality between obesity, T2DM, and metabolic syndrome is insulin resistance, the impaired response to insulin resulting in elevated levels of blood glucose [83,84].

In addition to the metabolic alterations, all three conditions are characterized by a chronic inflammatory state [85]. Interestingly, the circulating inflammatory cytokines are released from obese visceral WAT, and there, seemingly from infiltrating macrophages [86,87,88,89], which are localized to areas of adipocyte death [90]. Moreover, this chronic inflammation is intimately linked to the development of insulin resistance [88]. Macrophages are also found to a substantial proportion in the WAT of lean individuals where they are required for tissue surveillance and remodeling. Under physiological conditions, these macrophages are of the anti-inflammatory M2 subtype, whereas macrophages accumulating in obese WAT are of the pro-inflammatory M1 type [91,92]. However, treatment of obese mice with the M2-polarizing cytokine Interleukin 4 (IL-4) attenuated inflammation in adipose tissue and improved insulin sensitivity [93].

Finally, obesity is also characterized by oxidative stress [94] with an inverse correlation between central adiposity and antioxidant capacity [95]. Seemingly, ROS production increases selectively in adipose tissue, which was shown in obese mice and also in humans [96]. This goes along with a previous observation that adipocytes from mice fed an HFD produced approximately twofold more ROS than control adipocytes [97]. Moreover, in several cell models and obese, insulin-resistant mice, it was demonstrated that oxidative stress even serves as an important trigger for insulin resistance [98]. Not surprisingly, activation of NRF2, not only ameliorates obesity in mice on HFD [99,100], but also alleviates hyperglycemia [101] and insulin resistance [102], and reduces inflammation [100]. A link between obesity, oxidative stress, and inflammation was also found in a small cohort of obese patients. There it was shown that a single nucleotide polymorphism in the NLR family pyrin domain containing 3 (NLRP3) genes, which code for a component of the NLRP3 inflammasome complex, correlates with higher oxidative stress and an increase in inflammation [103].

On the organelle level, obesity and insulin resistance severely affect mitochondria. Oxidative stress has a direct impact on mitochondria as ROS inhibit mitochondrial respiration in white adipocytes and decrease mitochondrial membrane potential [104]. Moreover, high levels of glucose and fatty acids induce mitochondrial dysfunction in an adipocyte cell line in vitro [105]. However, mitochondrial functionality is essential for the synthesis of Adiponectin [106], an adipose tissue-derived anti-inflammatory hormone with favorable effects on insulin sensitivity. Therefore, mitochondrial dysfunction is intimately involved in adipose tissue inflammation and insulin resistance and improvement of mitochondrial functions could be of therapeutic value in obesity. Indeed, treatment of obese rats with MitoQ antioxidant acting on mitochondria prevents downregulation of Adiponectin [107]. In humans, it was shown that intake of the anti-diabetic drug Pioglitazone positively correlates with mitochondrial biogenesis in the subcutaneous fat of T2DM patients and the expression of mitochondrial enzymes involved in fatty acid oxidation [108], indicating that the beneficial effect on mitochondria may contribute to the lipid-lowering effects of the drug. Unfortunately, the loss of mitochondrial functions is not only observed in overt obesity but also at preclinical stages of acquired obesity, which was shown in a study in monozygotic twins [109].

In summary, all the above mentioned factors, many of which are common to obesity, T2DM, and metabolic syndrome, contribute to the enhanced risk for developing CVDs [110].

## 3. Protective Role of Curcumin in Cardiovascular Diseases

### 3.1. Effects of Curcumin on Cellular Senescence and Age-Related Cardiovascular Dysfunction

**Figure 1:**
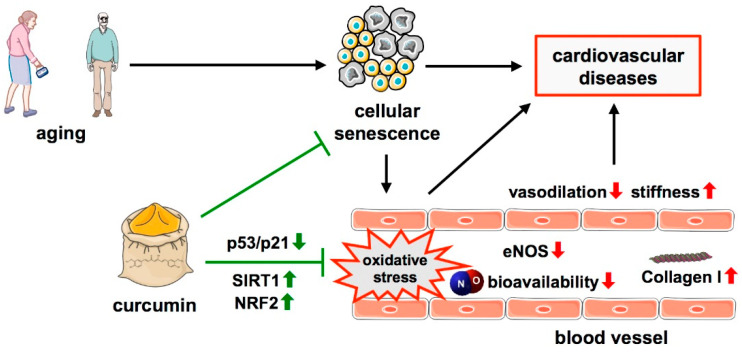
Protective effects of curcumin in age-related cellular senescence. A hallmark of aging, one major risk factor for cardiovascular diseases is cellular senescence which is associated with oxidative stress in blood vessels, along with decreased levels of eNOS, NO bioavailability, reduced vasodilation, and increased vascular stiffness due to increased Collagen I levels. Curcumin antagonizes these effects by the upregulation of SIRT1 and NRF2 and downregulation of the p53/p21 pathway. ↑—increased; ↓—decreased.

Curcumin can delay cellular senescence and reduce senescence-associated oxidative stress, both of which lead to vascular dysfunction. An in vitro study demonstrated that hydrogen peroxide-induced premature senescence in endothelial cells is attenuated by pre-treatment with curcumin for 24 h, along with a decrease in ROS production, and an increased eNOS activation and NO production [111] (Figure 1). With respect to age-associated endothelial dysfunction, it was shown that the dietary curcumin supplementation of old mice for 4 weeks led to improved vasodilation and a reduction in age-related large artery stiffness. These effects were ascribed to the restoration of NO bioavailability, reduction of vascular superoxide production and oxidative stress, as well as to a decreased Collagen I deposition [112] (Figure 1).

Based on these beneficial effects of curcumin in an animal model, efforts were focused on translating these findings to human subjects. In postmenopausal women, curcumin ingestion positively correlates with improved central arterial hemodynamics and reduced endothelial dysfunction [113,114]. Moreover, curcumin supplementation in healthy middle-aged and older adults is associated with an improvement in vascular endothelial function, underscoring the findings of the murine experiments [115]. The latter study indicates that curcumin might have preventive potential with respect to the age-related decline of vascular functions.

Some of the protective effects of curcumin have been attributed to the activation of Sirtuin 1 (SIRT1) [111]. Curcumin also counteracts senescence induction in rat hearts after administration of D-galactose (which induces oxidative stress and is widely used in animal aging models). There, it inhibits the p53/p21 signaling pathway by decreasing the expression of p53 and preventing oxidative stress [116] (Figure 1). Furthermore, curcumin activates NRF2, a major relay relevant in the protection against both oxidative stress and senescence, by multiple mechanisms [117] (Figure 1). An induction of NRF2 and an antioxidant response by curcumin was shown in primary rat neurons [118]. A similar response to curcumin was observed after oral application by gavage for 10 days in insulin-resistant mice where it improved glucose tolerance and reduced oxidative stress in muscle and liver by the upregulation of NRF2 [119].

The above studies provide evidence that curcumin may be a promising nutraceutical-based treatment for age-related CVDs. The anti-aging effects of curcumin are possibly mediated by its ability to delay senescence in the cells of the cardiovascular system via various pathways.

### 3.2. Effects of Curcumin on Adipose Tissue and Obesity

Apart from aging, obesity and the associated WAT dysfunction are major risk factors for the development of CVDs, and natural, plant-derived compounds have started gaining attention in novel, anti-obesity strategies. Moreover, not only a reduction of excessive WAT but also the beiging of white adipocytes provides an interesting approach towards reducing the risk for CVD development, which is also conferred by curcumin.

In vitro, in the preadipocyte cell line 3T3-L1, curcumin was shown to improve fatty acid oxidation while impairing lipogenesis, attenuate differentiation into mature adipocytes, and induce apoptosis in these cells [120,121]. This suggests that curcumin might be able to limit adipose tissue hyperplasia and hypertrophy which are initiating steps in obesity-induced WAT expansion.

**Figure 2:**
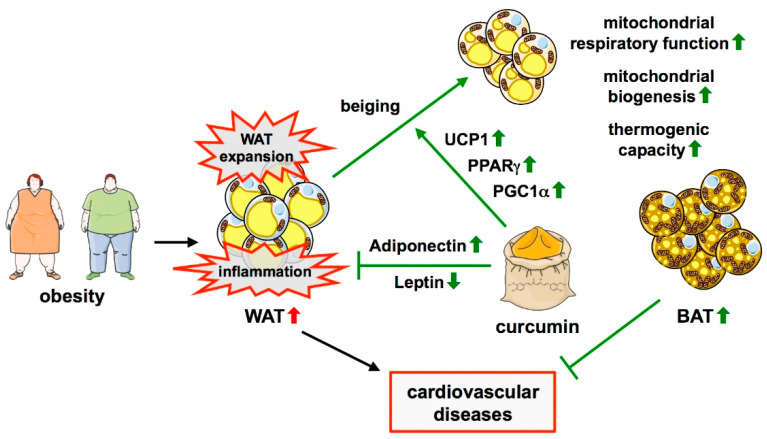
Protective effects of curcumin in obesity-induced adipose tissue dysfunction. Obesity is characterized by the expansion of WAT and inflammation therein. Curcumin can inhibit WAT expansion and obesity-induced adipose tissue inflammation. It is also linked to the process of beiging, the formation of beige adipocytes in WAT, which results in BAT-like characteristics of these cells. The underlying mechanisms include the upregulation of PPARγ, PGC1α, and UCP1, resulting in increased mitochondrial biogenesis, improved respiratory chain function, and thermogenesis. Moreover, curcumin induces an increase in Adiponectin levels with a concomitant decrease in Leptin, thereby reducing inflammation. ↑—increased; ↓—decreased.

In vivo, curcumin had profound effects on glucose handling, WAT function, and beiging. Although the regimen and duration of curcumin administration widely varied in the different mouse models used, several observations were common to all of them. In obese animals, no matter whether this condition was due to genetic manipulation or diet-induced, curcumin improved glycemic status and insulin sensitivity [120,122,123,124,125]. Moreover, inflammation in WAT was reduced as evident by—depending on the analyses—reduced macrophage accumulation [122,123,125,126] or a switch towards macrophages exhibiting an anti-inflammatory M2 polarization [124]. In all cases, this was associated with the reduced expression of proinflammatory cytokines. Thereby, curcumin can reduce obesity-induced adipose tissue inflammation, meaning that this natural compound could also be used for targeting inflammatory processes in the context of obesity (Figure 2).

In addition, curcumin can induce the beiging of white adipocytes, which was shown in 3T3-L1 cells, but more importantly, in the primary white adipocytes from rats [127] (Figure 2). Moreover, in some of the in vivo models, upregulation of UCP1 as a sign of beiging was observed with or without a cold stimulus [124,128] (Figure 2). Interestingly, studies in lean animals or cells derived from them showed divergent effects of curcumin on beiging in different WAT depots. Primary adipocytes isolated from rat inguinal WAT (iWAT) and treated with curcumin exhibited several features of brown adipocytes [127]. This matches observations made in mice where there were clear signs for induction of brown-like adipocytes in iWAT, whereas no such effect was observed in epididymal WAT (eWAT) [129,130]. The latter study also described an increase in M2 macrophages in this fat depot.

Several pathways have been implicated in the beiging effect of curcumin, which converge on improved mitochondrial functionality, going along with the high content and activity of these organelles in brown adipocytes. In 3T3-L1 cells and primary white adipocytes, curcumin induced upregulation of the transcription factor Peroxisome proliferator-activated receptor γ (PPARγ) and PPARγ coactivator 1α (PGC1α) [127] (Figure 2). These transcriptional regulators are capable of driving mitochondrial biogenesis and activation of the mitochondrial respiratory chain [131]. The upregulation of both was accompanied by an increase in mitochondrial density in the cell line as well as in primary adipocytes [127]. Another study showed that curcumin does not only upregulate PPARγ and PGC1α, but also increases mitochondrial respiration and ATP production in 3T3-L1 cells [128]. An effect of curcumin on mitochondrial biogenesis was also observed in mice where it increased PGC1α expression and mitochondrial DNA copy number in iWAT [129] (Figure 2).

Taken together, the above-mentioned studies show that curcumin positively affects the glycemic status and insulin sensitivity, promotes beiging of white adipocytes, and reduces obesity-associated adipose tissue inflammation. This suggests that curcumin might have therapeutic potential in the treatment of obesity.

There are only a few clinical trials in which the effects of curcumin in obese humans and subjects with metabolic syndrome or type 2 diabetes were assessed, however most of them had a very limited number of participants. A small randomized controlled trial on 44 overweight subjects with metabolic syndrome showed that curcumin administration is associated with reduced body fat and BMI [132]. A crossover trial with 30 obese subjects revealed a reduction in triglycerides and total HDL- and LDL-cholesterol, whereas body fat and BMI were not changed [133]. A randomized, placebo-controlled trial with 65 subjects with metabolic syndrome showed a decrease in triglycerides and LDL-cholesterol, and an increase in HDL-cholesterol, but no weight loss or changes in the glucose homeostasis [134]. Another such trial with 60 participants revealed a reduction in mean body weight and an improved pulse wave velocity (PWV), indicative of reduced vascular stiffness [135]. A larger study with 213 T2DM patients also found a reduction in PWV as well as lower triglyceride levels and less visceral and total body fat. In the same trial, Leptin levels were decreased, whereas Adiponectin was increased [136] (Figure 2). Intriguingly, the same changes in these hormones were detected in a meta-analysis of randomized controlled trials in patients with metabolic syndrome and related disorders [137]. The hormonal aspects are interesting in that Leptin effectively reduces food intake and body weight, whereas Adiponectin acts anti-hyperglycemic, anti-inflammatory, and anti-atherogenic. Although the correlations obtained in the clinical trials are incoherent—possibly due to different study designs—they suggest that curcumin might also be effective in obese humans or patients with metabolic syndrome or T2DM.

### 3.3. Protective Effects of Curcumin in Atherosclerosis

The possible atheroprotective properties of curcumin have been studied in many different animal models of atherosclerosis, including *Apoe*- and *Ldlr*-deficient mice or a combination of both, as well as by the use of atherogenic Western diets. Early studies in rabbits on an atherogenic diet revealed that a turmeric extract attenuates atherosclerosis development [138]. Similarly, curcumin administration was found to reduce the size of atherosclerotic lesions in a number of mouse models for atherosclerosis [139,140,141,142,143,144].

**Figure 3:**
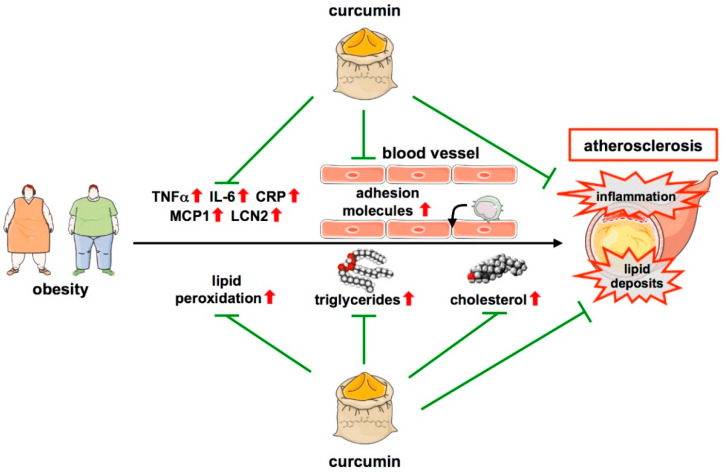
Protective functions of curcumin in atherosclerosis. Obesity is one major risk factor for atherosclerosis development. Atherosclerosis is characterized by low-grade inflammation with an increase in cytokines such as TNFα, IL-6, CRP, MCP1, and LCN2. Moreover, monocytes can infiltrate the vascular wall, another critical step in atherosclerosis development. By downregulating cytokines and reducing macrophage adhesion to the endothelium, curcumin attenuates inflammation. Another feature of atherosclerosis is lipid deposition in areas where atherosclerotic plaques develop, even long before an overt disease. This is fostered by lipid peroxidation as well as increases in serum triglycerides and cholesterol, all of which are attenuated by curcumin, which also leads to a favorable, non-atherogenic lipid profile reducing lipid deposition. ↑—increased; ↓—decreased.

This anti-atherogenic effect of curcumin could potentially be attributed to its ability to decrease the high plasma cholesterol levels and lipid peroxidation, both of which are fundamental features in the initiation of atherosclerosis, as well as to its capability to change the LDL- to HDL-cholesterol balance towards a more favorable, anti-atherogenic ratio (Figure 3).

Indeed, in atherosclerotic rabbits orally treated with different doses of curcumin, lower doses of the compound led to decreased susceptibility of LDL to peroxidation, along with reduced levels of plasma cholesterol, LDL-cholesterol, LDL-triglycerides, and LDL-phospholipids [145]. In *Ldlr* knockout mice on a high cholesterol diet, curcumin lowered the plasma levels of cholesterol, triglycerides, and LDL-cholesterol and increased HDL-cholesterol [140]. Similar observations were made in *Apoe*-deficient mice [144]. Furthermore, curcumin treatment in a rat coronary atherosclerosis heart disease model decreased the serum levels of triglycerides, total cholesterol, and LDL-cholesterol, while increasing HDL-cholesterol [146] (Figure 3). In one of these studies, it was also shown that curcumin induces transcriptional repression of HMG-CoA reductase, the rate-limiting enzyme in cholesterol synthesis [140]. With respect to the oxidative modification of lipids, it was shown more than two decades ago that a turmeric extract decreases oxidative stress in liver mitochondria in atherosclerotic rabbits, and thus, their susceptibility to lipid peroxidation [147] (Table 1).

In addition to the effects on lesion size, lipid profile, and oxidation, curcumin also affects inflammation, another hallmark of atherosclerosis, by reducing systemic levels of inflammatory cytokines such as IL-6, Tumor necrosis factor-alpha (TNFα), and C-reactive protein (CRP) [140,143,146] (Figure 3). A more detailed study revealed that these effects could be attributed to an upregulation of the Inhibitor of NFκB (IκB) in the aorta. This was supported by the fact that curcumin reduced DNA-binding and transcriptional activity of the pro-inflammatory transcription factor Nuclear factor kappa B (NFκB) in endothelial cells ex vivo [141]. Moreover, curcumin was shown to decrease the expression of the adipocyte-derived atherosclerotic marker Lipocalin 2 (LCN2), an inflammatory factor related to CVDs [143], and of Monocyte chemoattractant protein 1 (MCP1) in human vascular smooth muscle cells, another key inflammatory marker during the development of atherosclerosis [148] (Figure 3 and Table 1).

Another feature of atherosclerosis inhibited by curcumin is macrophage infiltration in atherosclerotic plaques. In the *Apoe*-deficient mouse model for atherosclerosis, curcumin reduced macrophage infiltration, probably through downregulation of adhesion molecules on endothelial cells, because it also inhibited monocyte adhesion to endothelial cells ex vivo [141]. This goes along with observations in rats, where curcumin reduced the permeability of the coronary artery. This effect was ascribed to inhibition of the expression of various cytokines, but also Matrix metalloproteinase (MMP) 9, which is synthesized by macrophages and plays a role in the degradation of the extracellular matrix [146]. Thereby, curcumin contributes to a delay in the formation of atherosclerotic plaques and to the stabilization of existing ones. Macrophages also play another critical role in atherosclerosis, as they turn into foam cells after scavenger receptor-dependent internalization of oxidized LDL. Interestingly, curcumin can suppress the expression of one of these receptors, namely CD36 [142] (Table 1).

The cumulative evidence from the animal studies described above clearly suggests that curcumin does not only positively affect major risk factors for CVDs but seems to play a role in reducing atherosclerosis development and progression.

### 3.4. Protective Effects of Curcumin in Myocardial Infarction

As for atherosclerosis, the impact of curcumin on MI and on changes occurring upon I/R injury has so far only been studied in animal models. The modalities of the experimental settings were vastly different, ranging from ex vivo experiments in isolated hearts over isoproterenol-induced cardiac injury to I/R injury after coronary artery ligation. Along the same lines, curcumin was used for different times in different doses and formulations, either as a pre-treatment prior to I/R or after ischemic injury. Nevertheless, several observations with respect to the protective effects of curcumin were common to all.

**Figure 4:**
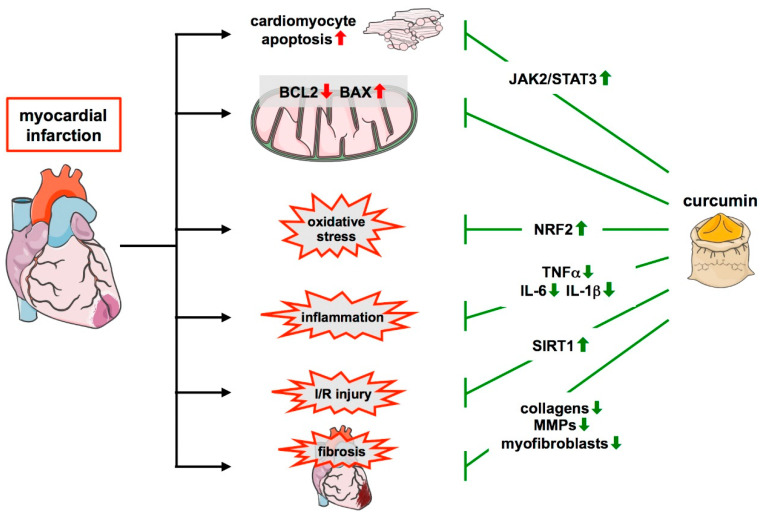
Protective role of curcumin in myocardial infarction and remodeling. During myocardial infarction, multiple changes occur in the infarcted heart which is positively affected by curcumin. This nutraceutical protects cardiomyocytes by activating the JAK2/STAT pathway and attenuates the unfavorable change in the levels of the apoptosis regulator BCL2 and BAX observed upon MI. Furthermore, it reduces oxidative stress via the upregulation of NRF2 and inflammation through the downregulation of cytokines such as TNFα, IL-6, and IL-1β. It also limits I/R injury, and this requires SIRT1. Additionally, it positively affects remodeling after infarction by reducing collagens and MMPs and suppressing myofibroblast overactivation, leading to a stable scar and preventing fibrosis. ↑—increased; ↓—decreased.

In isoproterenol-treated rats, which develop an infarct-like myocardial injury, curcumin attenuated morphological changes in the heart or even led to smaller injured areas [149,150]. A reduction in infarct size was observed in isolated hearts from curcumin-treated rats and mice subjected to I/R injury ex vivo [151,152,153]. The same protective effect of curcumin was seen in the analyses of hearts after I/R induced by coronary occlusion and reopening in mice and rats [154,155,156,157] (Figure 4 and Table 2).

On the one hand, curcumin protects cardiomyocytes against apoptosis, which was shown not only in a rat heart myoblast cell line but also in isolated neonatal cardiomyocytes under conditions of simulated I/R and cardiomyocytes in situ. Mechanisms involved include the upregulation of the anti-apoptotic apoptosis regulator BCL2 and downregulation of its counterplayer BCL2 associated protein X (BAX) [150,151,152,155,156,157,159,160] (Figure 4). The protection of cardiomyocytes was accompanied by normalization of serum creatine kinase and lactate dehydrogenase levels upon curcumin administration in these models, both of which are indicators of tissue damage and are elevated after MI [149,150,151,152,157,158] (Table 2).

With respect to remodeling processes after MI, it has been shown that curcumin prevents upregulation of the expression of various collagens, collagen deposition, the overactivation of matrix metalloproteinases, and the persistence of large numbers of myofibroblasts. Thereby, it positively affects remodeling and effectively reduces scar size [154,156] (Figure 4). In line with these findings, curcumin also attenuates the decline in heart functions after MI. This became obvious from improvements in, e.g., left ventricular ejection fraction, contractility, or faster recovery of mechanical work [149,151,152,153,154,156,158,159,161,162]. These studies demonstrate that curcumin can effectively counteract maladaptive cardiac repair and preserve cardiac function after MI (Table 2).

Moreover, curcumin also has a profound effect on inflammation after I/R as it can attenuate the upregulation of inflammatory cytokines such as TNFα, IL-1β, or IL-6, and the infiltration of inflammatory cells in the infarcted area [153,161] (Figure 4). In addition, curcumin has an influence on macrophage polarization in I/R. After ischemia and 7 days of reperfusion in mice, curcumin decreased the expression of markers for M1 macrophages and increased markers indicative of the M2 subtype in the myocardium. Flow cytometric analyses then confirmed the suppression of M1 cells and enhanced expansion of M2 cells [161] (Table 2).

Additionally, curcumin plays an important role in re-establishing redox homeostasis. Whenever examined in these models, I/R injury was accompanied by a decrease in GSH and reduced activity of anti-oxidative enzymes such as Catalase and SOD, as well as an increase in lipid peroxidation. In all these cases, curcumin could attenuate or sometimes block the occurrence of oxidative stress [149,150,152,154,155,156,158,159,162]. Although most enzymatic measurements did not differentiate between isoforms of anti-oxidative enzymes, one study clearly showed a curcumin-induced increase in RNA and protein levels of NRF2, which upregulates the expression of SOD2, the mitochondrial superoxide dismutase [155]. A direct effect on mitochondria was demonstrated by the observation that curcumin improves mitochondrial membrane potential in rat heart myoblasts undergoing simulated I/R [156] and enhances respiration in mitochondria isolated from hearts subjected to I/R ex vivo [162] (Table 2).

The aforementioned cardioprotective effects of curcumin in the setting of MI can be exerted through many different pathways. In addition to effects on antioxidative defense systems such as NRF2 and SOD2 and the mitochondrial respiratory chain, another pathway activated by curcumin affects the mitochondria. In neonatal cardiomyocytes, isolated hearts, and in vivo I/R it was shown that the histone deacetylase SIRT1, which plays a vital role in metabolic control, mitochondrial functions, biogenesis, and turnover [163], is downregulated after I/R, but could be reactivated by curcumin pretreatment. That the associated protective effects of curcumin in these models were SIRT1-dependent, was shown by the use of the SIRT1 inhibitor sirtinol and knockdown of SIRT1 expression [152] (Figure 4 and Table 2).

SIRT1 has also been shown to induce autophagy [164], a process critical for cellular proteostasis, especially in post-mitotic tissues, owing to their limited proliferative capacity. Consistently, defects in autophagy exacerbate the development of cardiovascular diseases [165]. Moreover, SIRT1 is also upregulated by caloric restriction (CR) [166], one of the most effective nutritional interventions with a positive impact on cardiovascular health [167]. CR—similar to SIRT1—has been demonstrated to induce autophagy [168]. Mechanistically, CR suppresses the mammalian target of the rapamycin (mTOR) pathway [169] and phosphorylation of CREB binding protein (CBP)/p300 by mTOR complex 1 (mTORC1), which is required for autophagy inhibition [170]. Thus, CR counteracts the detrimental effects of permanent mTORC1 activation, which is observed in many metabolic and cardiovascular disorders [171]. Several natural substances including curcumin have been shown to evoke similar effects as CR and, therefore, have been termed caloric restriction mimetics [172,173]. Curcumin is a specific CBP/p300 inhibitor [174] and can also induce autophagy. This has been demonstrated ex vivo in different cardiovascular cell types such as endothelial cells [175,176,177], vascular smooth muscle cells [178], and cardiomyocytes [179]. More importantly, in vivo, curcumin ameliorates the outcomes after experimental myocardial infarction by upregulating autophagy and protecting the mitochondrial function [156]. The latter observation leads to the speculation that curcumin might also have an impact on mitophagy, an organelle-targeted form of autophagy removing damaged mitochondria [180]. Indeed, it was shown in a rat model that curcumin protects against cerebral I/R injury by regulating mitophagy [181].

Apart from the aforementioned regulators of mitochondrial functions, another pathway that has received attention in the context of protective effects of curcumin in MI involves the Janus kinase 2 (JAK2) and its downstream target Signal transducer and activator of transcription 3 (STAT3) (Figure 4). The importance of STAT3 in MI had already been demonstrated in transgenic mice with cardiomyocyte-specific overexpression of this transcription factor in which the infarct size and ROS generation were lower than in non-transgenic littermates [182]. When the JAK2/STAT3 pathway was blocked with the JAK2 inhibitor AG490, the positive effects of curcumin on infarct size in non-transgenic mice and rats such as cardiomyocyte apoptosis, ROS generation, and post-ischemic functional recovery of the heart were abrogated [151,156]. STAT3 is interesting in that it seemingly plays a role in cardioprotection by so-called remote ischemic conditioning (RIC), repeated brief episodes of I/R in limbs. This maneuver has been shown to be cardioprotective in clinical studies [183] and leads to improved mitochondrial function, as shown in coronary artery bypass graft patients [184]. In a pig model, RIC reduces infarct size and leads to activation of STAT3 in the myocardium and better preservation of mitochondrial complex I respiration. All these effects were abrogated by inhibition of JAK2 or STAT3 itself [185]. Increased STAT3 phosphorylation in the anterior wall of the heart has also been shown in mice subjected to RIC. Importantly, the reduction in infarct size of RIC was lost in aged animals and also in young mice treated with AG490 or with a cardiomyocyte *stat3*-deficiency [186]. In summary, these observations suggest that STAT3 plays an important role in cardioprotection by RIC and that the activation of STAT3 by curcumin might have similar effects (Table 2).

Several of the above studies have investigated the effect of curcumin treatment after the induction of ischemia and only during the reperfusion period and could show that it is still cardioprotective beyond a preventive use [151,154,160,161] (Table 2). This could make curcumin an interesting option as a supportive therapy after ischemia.

## 4. Future Directions

Curcumin can positively affect different risk factors for CVDs and the outcome in the diseases themselves which has been shown in a number of animal models. More importantly, it is effective against cardiovascular diseases and has shown a promising impact on cardiovascular diseases in clinical studies [187]. Unfortunately, the low aqueous solubility and absorption in the gastrointestinal tract of free curcumin result in a poor pharmacokinetic profile and bioavailability. However, simply increasing the dose does not necessarily lead to a higher serum concentration in humans [188]. Therefore, over the years, different approaches have been pursued to improve the bioavailability of curcumin. Already more than 20 years ago, it was shown that piperine, an alkaloid responsible for the pungency of black pepper, dramatically increases the serum levels of curcumin in rats and humans [189]. Later, similar effects were observed with Ilepcimide, a synthetic piperine analog in rats [190]. In addition to using other natural substances as enhancers of curcumin’s bioavailability, a number of different formulations have been shown to improve its uptake and stability. These include dispersions with various carriers, micronized powders, as well as nanoparticle and micellar formulations [191,192]; some of these formulations exhibit a greatly enhanced bioavailability in humans [193]. It will be interesting to systematically compare these various application forms in in vivo models with respect to their effectiveness regarding outcomes on age- and obesity-related processes fostering the development of CVDs. The most promising formulations then need to be tested in well-designed clinical studies to develop curcumin derivatives as therapeutic drugs.

Moreover, other natural compounds, which are regularly consumed or approved as dietary supplements, have been shown to reduce the mortality of CVDs or risk factors for their development. Two large epidemiological studies with more than 700,000 participants demonstrated that coffee drinking was inversely correlated with subsequent mortality, including death from heart disease and stroke [194,195]. A recent machine learning analysis of several studies found that higher coffee intake was associated with a reduced risk of heart failure [196]. Ingredients of another widely consumed beverage, flavanols, polyphenolic compounds in cocoa, also reduce the risk for CVDs [197,198]. In addition, TA-65, a small molecule from the medicinal plant *Astragalus membranaceus* used in traditional Chinese medicine, given over a five-year period, reduces fasting glucose, total and LDL-cholesterol, as well as blood pressure, all risk factors for CVDs [199]. It is captivating to speculate that these plant-derived molecules affect different cellular pathways, and thus, might potentially act additively or even synergistically in reducing the risk for CVDs.

## Figures and Tables

**Table 1:** Curcumin effects in animal models for atherosclerosis.

Model	Curcumin Dose and Application Route	Curcumin Effects	Ref.
New Zealand rabbits on HFD	1.66 and 3.2 mg/kg bw turmeric hydroalcoholic extract (10% curcumin) oral for 7 weeks	intracellular membrane lipid peroxidation ↓	[147]
New Zealand rabbits on HFD	1.66 and 3.2 mg/kg bw turmeric hydroalcoholic extract (10% curcumin) oral for 7 weeks	total cholesterol ↓ LDL-cholesterol ↓ LDL-triglycerides ↓ LDL-phospholipids ↓ LDL lipid peroxidation ↓	[145]
New Zealand rabbits on HFD	1.66 mg/kg bw turmeric hydroalcoholic extract (10% curcumin) oral for 10, 20, and 30 days	lesion size ↓ plasma lipid peroxidation ↓	[138]
Wistar rats on HFD plus intraperitoneal vitamin D3 injection	100 mg/kg bw curcumin per day via oral gavage for 4 weeks	triglycerides ↓ total cholesterol ↓ LDL-cholesterol ↓ HDL-cholesterol ↑ arterial permeability ↓ MMP9 ↓ TNFα, CRP ↓	[146]
C57BL/6J x 129/SvJ *Apoe*^−/−^ *Ldlr*^−/−^ mice on HFD	0.3 mg/day curcumin in chow for 16 weeks	lesion size ↓	[139]
C57BL/6J *Ldlr*^−/−^ mice on HFD	500, 1000, and 1500 mg/kg bw curcumin in chow for 16 weeks	lesion size ↓ IL-6, MCP1 ↓ CD36 ↓	[142]
C57BL/6J *Ldlr^−/−^* mice on HFD	0.02% (*w*/*w*) curcumin in chow for 18 weeks	lesion size ↓ triglycerides ↓ total cholesterol ↓ LDL-cholesterol ↓ HDL-cholesterol ↑ HMG-CoA reductase ↓ CRP ↓	[140]
C57BL/6J *Apoe*^−/−^ mice on HFD	0.1% (*w*/*w*) curcumin in chow for 16 weeks	lesion size ↓ total cholesterol ↓ LDL-cholesterol ↓	[144]
C57BL/6J *Apoe*^−/−^ mice on HFD	0.2% (*w*/*w*) curcumin in chow for 16 weeks	lesion size ↓ macrophage infiltration ↓ IκB ↑	[141]
C57BL/6J *Apoe*^−/−^ mice on HFD	40, 60 and 80 mg/kg bw curcumin per day via oral gavage for 12 weeks	lesion size ↓ triglycerides ↓ total cholesterol ↓ LDL-cholesterol ↓ TNFα, CRP, IL-6, LCN2 *↓*	[143]

↑—increased; ↓—decreased.

**Table 2:** Curcumin effects in animal models for myocardial infarction. Only models in which I/R was performed in vivo are listed.

Model	Curcumin Dose and Application Route	Curcumin Effects	Ref.
Wistar rats subcutaneous isoproterenol injection	25, 50, 100, 200 mg/kg curcumin per day via oral gavage starting 2 days before isoproterenol	heart function ↑ CK, LDH ↓ anti-oxidative enzymes ↑ lipid peroxidation ↓	[149]
Wistar rats subcutaneous isoproterenol injection	50 mg/kg bw curcumin per day via oral gavage for 9 days starting with isoproterenol	scar size ↓ CK, LDH ↓ apoptosis ↓ SOD ↑ oxidative stress ↓	[150]
Wistar-Bratislava rats subcutaneous isoproterenol injection	100, 150 and 200 mg/kg bw curcumin per day via oral gavage for 15 days starting with isoproterenol	heart function ↑ CK, LDH ↓ oxidative stress ↓	[158]
Sprague–Dawley rats LAD ligation	200 mg/kg bw curcumin per day via oral gavage starting 10 days before MI	infarct size ↓ CK, LDH ↓ BCL2 ↑ BAX ↓ SIRT1 ↑	[152]
Sprague–Dawley rats LAD ligation	10, 20 and 30 mg/kg bw curcumin per day via oral gavage starting 20 days before MI	infarct size ↓ heart function ↑ BCL2 ↑ BAX ↓	[159]
Sprague–Dawley rats LAD ligation	25, 50 and 100 mg/kg bw curcumin single intraperitoneal bolus injection 30 min before MI	infarct size ↓ CK, LDH ↓ SOD, GSH ↑ BCL2 ↑ BAX ↓ oxidative stress ↓	[157]
Sprague-Dawley rats LAD ligation	20 µL 40 μM curcumin or curcumin hydrogel injected into the ventricular wall during ischemia	infarct size ↓ heart function ↑ apoptosis ↓ anti-oxidative enzymes ↑ phospho-JAK2/STAT3 ↑	[156]
C57BL/6 mice LAD ligation	100 mg/kg bw curcumin or 10 mg/kg bw curcumin analog 14p per day via oral gavage starting 7 days before MI	infarct size ↓ CK ↓ apoptosis ↓ BCL2/BAX ratio ↑ oxidative stress ↓ NRF2 ↑	[155]
Sprague-Dawley rats LAD ligation	150 mg/kg bw curcumin per day in peanut paste for 7, 21, or 42 days starting after ischemia	infarct size ↓ heart function ↑ scar size ↓ MMP2, MMP9 ↓ Collagen I, III, IV ↓ myofibroblasts ↓	[154]
Sprague-Dawley rats LAD ligation	150 mg/kg bw curcumin per day in peanut paste for 28 days starting after ischemia	apoptosis ↓ BCL2 ↑ NFκB p65 *↓*	[160]
C57BL/6 mice LAD ligation	100 mg/kg/day curcumin injected intraperitoneally for 6 weeks starting after ischemia	heart function ↑ fibrosis ↓ Collagen I ↓ TNFα, IL-6, IL-1β ↓ M1 macrophages ↓ M2 macrophages ↑	[161]

↑—increased; ↓—decreased.
